# Correction: Montalesi et al. Divergent Effects of Daidzein and Its Metabolites on Estrogen-Induced Survival of Breast Cancer Cells. *Cancers* 2020, *12*, 167

**DOI:** 10.3390/cancers16233890

**Published:** 2024-11-21

**Authors:** Emiliano Montalesi, Manuela Cipolletti, Patrizio Cracco, Marco Fiocchetti, Maria Marino

**Affiliations:** Department of Science, University Roma Tre, Viale Guglielmo Marconi 446, I-00146 Roma, Italy; emiliano.montalesi@uniroma3.it (E.M.); manuela.cipolletti@uniroma3.it (M.C.); patrizio.cracco@uniroma3.it (P.C.); marco.fiocchetti@uniroma3.it (M.F.)

In the original publication [1], there was a mistake in Figure 4a as published. During figure preparation, we mistakenly included the same line twice (total AKT) in Figure 4a instead of the tubulin present in the original figures. The corrected Figure 4a appears below. In addition, the email of the author Patrizio Cracco has also been changed. The authors apologize for any inconvenience caused and state that the scientific conclusions are unaffected. This correction was approved by the Academic Editor. The original publication has also been updated.

## Figures and Tables

**Figure 4:**
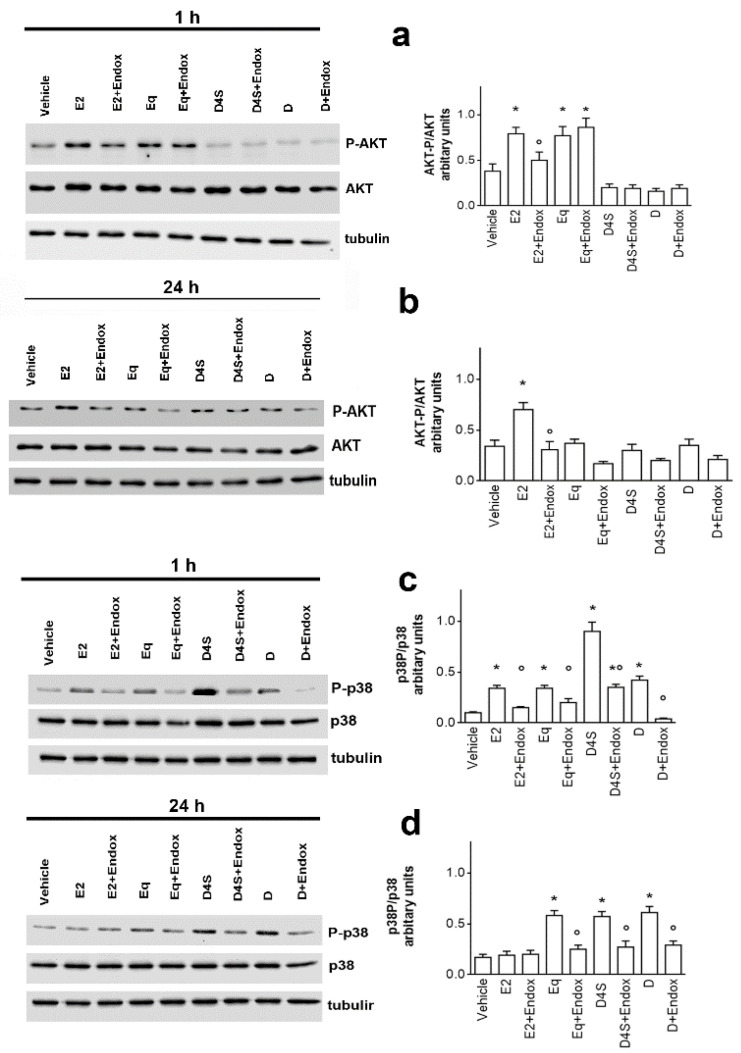
Daidzein, daidzein-4′-sulfate and equol action mechanism. The phosphorylation of the Ser473 residue of AKT (pAKT) (**a**,**b**) and Thr180/Tyr182 residues on P-38 (**c**,**d**) was determined by western blot analysis in MCF-7 cells exposed for 1 h (**a**,**c**) and 24 h (**b**,**d**) to either vehicle (DMSO:PBS 1:1) or E2 (10 nM) in presence or absence of D, D4S and Eq (1 μM). The nitrocellulose was stripped and then probed with anti-AKT or anti-p38 antibodies. In the panels, the PAKT/AKT (**a**,**b**) and Pp38/p38 (**c**,**d**) ratios are represented. These ratios are calculated with respect to tubulin obtained by densitometric analyses of three different experiments (mean ± SD). *p* < 0.001 was determined by Student t-test with respect to vehicle (*) or Endox-untreated (°) samples. AKT: protein kinase B; E2: estradiol; Endox: endoxifen; ERα: estrogen receptor α; NGB: neuroglobin; p38: p38 mitogen-activated protein kinase; D: daidzein; D4S: daidzein-4′-sulfate; Eq: equol.
